# Social loneliness and internet gaming addiction among adolescents: the mediating roles of escape motivation and self-control

**DOI:** 10.3389/fpsyg.2026.1793599

**Published:** 2026-05-29

**Authors:** Qiying Gan, Hong Wang

**Affiliations:** Faculty of Teacher Education, Wenshan University, Wenshan, Yunnan, China

**Keywords:** Compensatory Internet Use Theory, escape motivation, internet gaming addiction, self-control, social loneliness

## Abstract

**Background:**

In recent years, Internet gaming addiction among adolescents has become increasingly severe, emerging as a pressing global mental health concern. Much of the existing literature has concentrated on factors such as personality, family backgrounds, and the intrinsic appeal of video games, yet the psychological underpinnings of gaming addiction remain underexplored. This research aims to examine how feelings of social loneliness contribute to gaming addiction among teens, focusing on how motivations to escape reality and the ability to regulate one’s actions serve as intermediaries in this relationship.

**Methods:**

Drawing on the Compensatory Internet Use Theory, this cross-sectional study surveyed 731 middle school students using validated scales measuring social loneliness, Internet gaming addiction, escape motivation, and self-control. Hypotheses were tested using SPSS 27.

**Results:**

The results showed a significant positive association between social loneliness and Internet gaming addiction in adolescents. In addition, escape motivation and self-control jointly functioned as mediating variables in the association between social loneliness and gaming addiction.

**Conclusion:**

This research elucidates the psychological mechanisms linking social loneliness to internet gaming addiction among adolescents, highlighting the critical mediating roles of escape motivation and self-control. The findings provide theoretical backing for efforts aimed at preventing and intervening in adolescent Internet gaming addiction. They highlight the need for educators and parents to closely monitor adolescents’ escape motivation and self-control abilities to mitigate the risk of addiction to gaming.

## 1 Introduction

Rapid developments in smartphone and internet technologies have transformed adolescent entertainment, with Internet gaming now occupying a central role in daily life (Çelikkaleli et al., 2025). This shift has made adolescents particularly susceptible to internet gaming addiction (IGA). IGA is defined as the overuse and lack of control over online gaming, which can lead to physical, psychological, and social impairments (Block, 2008). As a typical manifestation of excessive immersion in online gaming, IGA serves as a critical indicator for assessing digital health risks among adolescents (Gioia et al., 2022; Kuss et al., 2021). In China, the prevalence of IGA among adolescents is particularly pronounced, ranging from 2.2 to 21.5%, such prevalence is well above that reported for peers in these Western regions, ranging from 1.4 to 9.9% (Quancai et al., 2023). Studies have shown that IGA not only severely undermines adolescents’ academic performance but also has profound negative effects on their mental health (Abbas, 2022). Therefore, investigating the causes and mechanisms underlying adolescent IGA is crucial for designing successful prevention and intervention measures.

Existing research has identified factors such as autism spectrum disorder, social bullying, sleep quality, attention deficits, and social loneliness (SL) as being associated with IGA among adolescents (Berloffa et al., 2022; Eltahir et al., 2025; Hammad et al., 2024; Nwanosike et al., 2022). Among these factors, adolescence represents a particularly sensitive period for the experience of SL, an emotional state regarded as a significant psychological contributor to IGA in this age group (Gan et al., 2022; Haberlin and Atkin, 2022). Furthermore, escape motivation (EM) and self-control (SC) are also key variables for understanding adolescent IGA. Prior studies have demonstrated that stronger EM and weaker SC increase the risk of IGA among adolescents (Carmona and Whiting, 2021; Gu and Mao, 2023; Yu et al., 2023).

Although numerous studies have examined how SL is associated with adolescents’ online gaming behaviors, research on its relationship with EM and SC remains limited. In particular, existing literature has provided limited insight into the mechanisms by which SL is indirectly associated with IGA through its links with EM and SC. To bridge this gap in research, the study at hand, grounded in the Compensatory Internet Use Theory (CIUT), constructs a model encompassing SL, EM, SC, and IGA. The study seeks to clarify how SL is associated with IGA in adolescents, thereby providing empirical evidence to inform prevention and intervention efforts targeting this pressing issue.

## 2 Theoretical underpinning and hypotheses

### 2.1 Theoretical underpinning

As outlined by the CIUT, individuals often use the Internet as a means to fill the gaps or unmet needs in their real-world experiences, particularly when confronted with negative emotions and stressors. In such circumstances, the Internet offers a means of escape (Kardefelt-Winther, 2014). This theory has been extensively validated in research on digital behaviors and provides an important explanatory framework for understanding adolescent IGA (Fu et al., 2020). When adolescents experience strong feelings of SL in real life, the immersive experience and instant rewards offered by Internet games serve as a powerful compensatory mechanism that meets their unmet social needs (Quancai et al., 2023). Research suggests that individuals who face social isolation in their everyday lives tend to seek refuge online, as the internet provides temporary relief from negative emotions (Fan et al., 2022). Nevertheless, this compensatory usage is fundamentally a maladaptive coping mechanism; depending on it to control unpleasant feelings frequently compromises SC, resulting in overuse and, eventually, addicted behaviors (Liu et al., 2024). Therefore, adopting the CIUT as the theoretical framework enables a clear explanation of how SL is associated with adolescent IGA through its links with EM and SC. This model presents a novel way to comprehend the psychological dynamics driving adolescent addiction and serves as a solid theoretical foundation for crafting intervention methods.

### 2.2 Hypotheses

#### 2.2.1 Social loneliness and internet gaming addiction

SL is conceptualized as the perceived lack of broader social networks and peer interactions, distinct from emotional loneliness, which reflects the absence of close attachment relationships (Weiss, 1973). SL has been found to be positively associated with IGA in adolescents, suggesting that greater feelings of social isolation may increase vulnerability to problematic gaming behaviors (Bal and Balcı, 2026; Dong et al., 2024; Mun and Lee, 2022). Notably, these findings pertain specifically to adolescent populations, highlighting the developmental relevance of social experiences at this age. For example, Yu et al. (2022) found that socially lonely adolescents increased their gaming time to cope with negative emotions and maintain social connections in virtual environments. Other studies with adolescent samples have similarly found that higher SL is linked to greater risk of problematic gaming (Mihaliková and Dedová, 2024; Yu et al., 2023). From the perspective of the CIUT, adolescents who are suffering SL may use gaming as a coping mechanism to get away from unpleasant feelings or social difficulties in real life, which directly contributes to the development of IGA (Kardefelt-Winther, 2014). These results suggest that SL is a key psychosocial risk factor for adolescent IGA. Based on this evidence and theoretical rationale, the present study proposes:

*H1*: SL is significantly and positively associated with IGA.

#### 2.2.2 The mediating role of escape motivation

In the context of online games, EM is defined as the inclination of individuals to intentionally engage in Internet gaming as a means of escaping from the real world or avoiding problems in their social lives (Caplan et al., 2009). Research indicates a positive link between SL and EM among adolescents (Chen et al., 2023). Recent evidence also emphasizes that the interplay between parental mediation, social skills, and escape-driven gaming is developmentally specific to adolescence, highlighting that these processes may operate differently than in adults (Commodari et al., 2024). Negative emotions such as SL can lead individuals to form negative expectations about interpersonal interactions, thereby prompting them to avoid real-life social engagement (Wei et al., 2023). To demonstrate, Li J. et al. (2021) found that EM is a product of loneliness; the loneliness stemming from unsatisfactory social interactions activates EM in adolescents. Moreover, individuals experiencing SL are more prone to developing altered views of both their own identity and the world around them, resulting in strong dissatisfaction with real life (Zhen et al., 2019). As outlined by the CIUT, such dissatisfaction may drive individuals toward the virtual world, reducing interactions with others in the real world and fostering stronger EM (Kardefelt-Winther, 2014). These findings suggest that SL is an important predictor of adolescents’ EM. However, research on the link between SL and EM remains limited, making further investigation essential to understanding the psychological mechanisms underlying adolescent IGA.

Research indicates a positive link between EM and IGA among adolescents (Hellström et al., 2012; Melodia et al., 2022). Within the realm of Internet gaming, adolescents’ EM manifests as a desire to temporarily flee from negative emotional states through highly immersive gaming experiences (Cudo et al., 2022; Myrseth et al., 2017; Nebel and Ninaus, 2022). Specifically, Liao et al. (2020) reported that among Chinese adolescent gamers, escape motivation was positively associated with Internet gaming engagement and gaming disorder, indicating that adolescents often play games to temporarily avoid negative emotions or real-life stressors. In addition, individuals experiencing SL often have highly negative experiences in their real-life interpersonal relationships and might resort to the Internet to escape this sense of isolation, which can contribute to IGA (Fernandes et al., 2020; Lopez-Fernandez et al., 2018; Öz and Üstün, 2022). For example, Slack et al. (2022) reported that socially lonely individuals are more motivated to engage in game-related virtual communities to make up for the absence of real-life social connections and support. In summary, SL may be directly associated with IGA and may also be indirectly linked to it through higher levels of EM. Building on this, the study formulates the following hypothesis:

*H2*: EM acts as a mediator between SL and IGA.

#### 2.2.3 The mediating role of self-control

SC refers to the regulation of cognition, emotion, and behavior to achieve long-term goals (Tangney et al., 2004). Research has shown that SL is negatively associated with individuals’ levels of SC (Jeon et al., 2022; Jeong et al., 2019). This is because the experience of SL depletes the psychological resources necessary for successful SC, and SC resources are limited; when these resources are exhausted, individuals’ ability to regulate their behavior is weakened (Campbell et al., 2006; Stavrova et al., 2022). For instance, Li X. W. et al. (2021) found that among adolescents, negative emotions such as SL consume psychological resources, leading to increased boredom and reduced self-control. Furthermore, adolescents experiencing long-term SL are more prone to favoring immediate gratification and show significantly reduced persistence in pursuing long-term goals, further damaging their SC resources (Yue et al., 2022). Based on this analysis, it can be inferred that SL undermines adolescents’ SC.

Research has shown a significant negative association between SC and IGA among adolescents (Cudo et al., 2020; Jing et al., 2026). Specifically, higher SC in adolescents is associated with improved regulation of irrational cognitions, emotions, and behaviors. As a result, they manage maladaptive online behaviors more effectively and show reduced reliance on the Internet, thereby significantly lowering the risk of IGA (Jeon et al., 2022; Quancai et al., 2023). For example, Phetphum et al. (2023) found that those with higher SC tend to manage their emotions effectively and exhibit a greater sense of responsibility, resulting in a lower prevalence of IGA. In addition, the more SL adolescents experience, the stronger their motivation to fulfill emotional needs, while their SC may be further diminished (Yue et al., 2022). For example, college student research has shown that SL can reduce SC, increase the gratification derived from Internet gaming, and consequently heighten the risk of addiction (Guo et al., 2024), suggesting a similar mechanism may operate in younger populations. In summary, SL may be directly associated with IGA and may also be indirectly linked to it through lower levels of SC. Building on this, the present research formulates the following hypothesis:

*H3*: SC acts as a mediator between SL and IGA.

### 2.3 Current research

**Figure 1:**
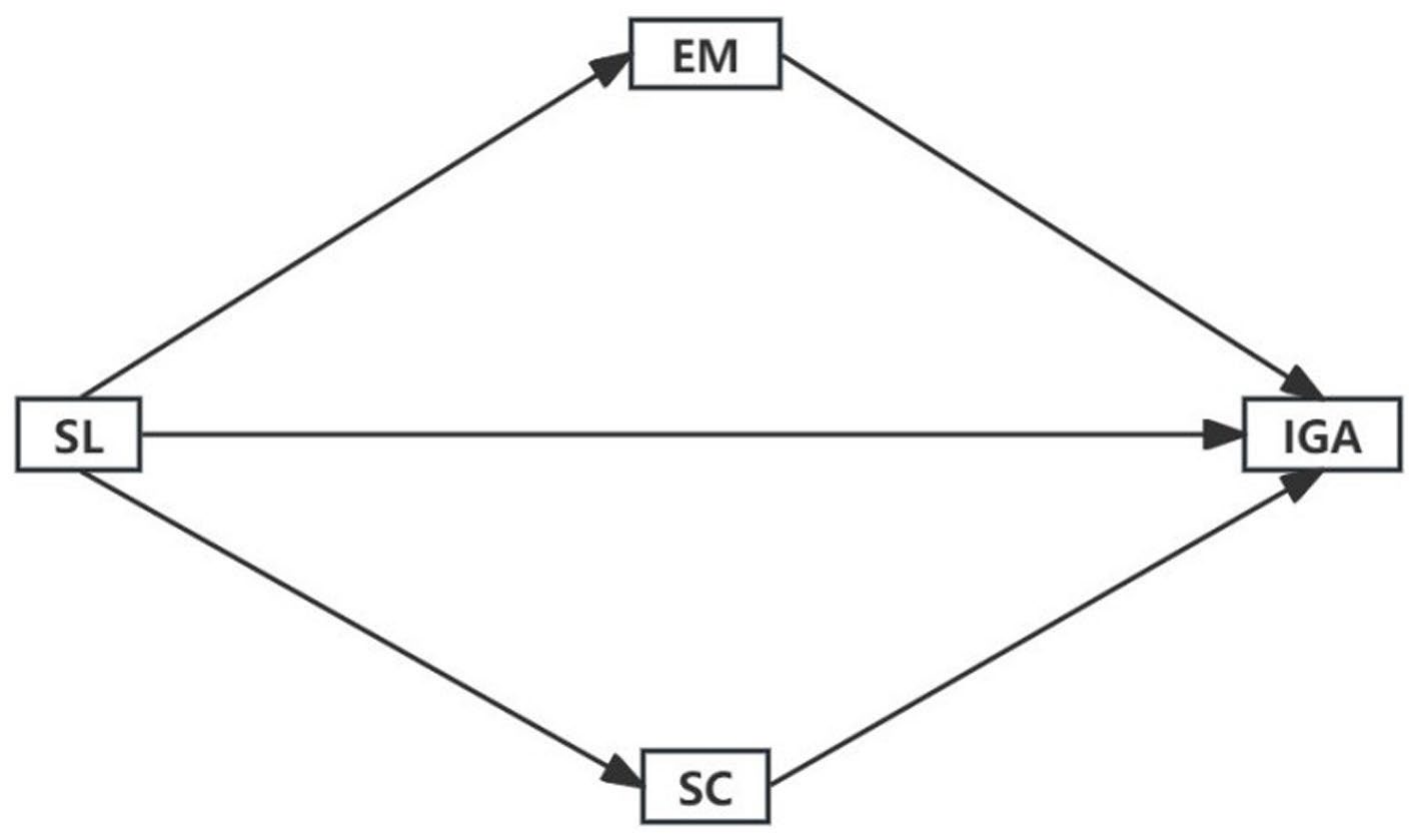
Research model.

This study examined the association between SL and IGA among adolescents, focusing on EM and SC as parallel mediators. Guided by the CIUT framework, a mediation model (Figure 1) was constructed to explore whether these pathways explain the SL–IGA link.

## 3 Methods

### 3.1 Participants

This study employed a multi-school questionnaire survey design for questionnaire distribution and data collection, and the survey was administered via the online platform Wenjuanxing.^1^ Ethical approval was granted by the Wenshan University Ethics Committee, and data were collected from October to November 2025. Middle school students were recruited from several schools in China through school-based coordination and online questionnaire distribution. Rather than claiming national representativeness, this study aimed to include students from different school contexts to enhance sample heterogeneity. However, detailed information regarding the number of participating schools, geographic regions, urban–rural distribution, and socioeconomic composition was not fully recorded during data collection. Thus, the generalizability of the results warrants caution. Before participation, all participants were briefed on the study and guaranteed confidentiality. It was completely voluntary to participate, and informed consent was obtained in accordance with ethical requirements.

In accordance with the sample size estimation guidelines provided by Kline (2018), structural equation modeling requires at least 10 participants per item, with an additional 20% allowance for potential sample loss. Given that the questionnaire contained 50 items, the estimated required sample size was 600 participants (50 × 10 × 1.2). A sum of 774 questionnaires was obtained, with 43 deemed invalid, resulting in 731 usable responses. The effective response rate was 94%, indicating a sufficient sample size and good data quality to support model construction and statistical inference. To minimize bias from ceiling and floor effects, surveys were excluded if (1) over 20% of items were unanswered or (2) more than 80% of responses were extreme (Dong et al., 2012).

The majority of participants were between 14 and 17 years old (accounting for 89.19% of the sample). Regarding gender distribution, there were 427 males (58.41%) and 304 females (41.59%), reflecting a relatively balanced and representative group (Table 1).

**Table 1:** Demographic information of participants.

Variable	Count	Percentage
Gender		
Male	427	58.41%
Female	304	41.59%
Age		
13 years old or below	42	5.75%
14–15 years	313	42.82%
16–17 years	339	46.37%
18 years old or above	37	5.06%
Grade		
Junior high school	357	48.84%
High school	374	51.16%
Family economic status		
Poor	99	13.54%
Average	536	73.33%
Good	96	13.13%
Place of residence		
Urban	357	48.84%
Rural	374	51.16%
Only child		
Yes	310	42.41%
No	421	57.59%

To control for potential demographic confounds, differences in IGA levels were compared across key demographic groups (see Table 2). Analysis indicated that IGA did not vary significantly with age, academic grade, or family socioeconomic background (all *p* > 0.05). In contrast, a statistically significant difference emerged between genders (*p* < 0.05), leading us to include gender as a covariate in all subsequent analyses.

**Table 2:** Variations in IGA by demographic factors.

Demographic variables	*t*	*F*	*p*
Gender	4.738		<0.001
Grade	0.845		0.399
Only child	1.085		0.278
Place of residence	1.724		0.085
Age		1.567	0.196
Family economic status		2.170	0.115

### 3.2 Measures

#### 3.2.1 Social loneliness scale (SLS)

This research employed the SLS developed by Hays and DiMatteo (1987) to assess adolescents’ experiences of SL. There are eight items in the scale, including two reverse-scored items (e.g., “When I need companionship, I can find a friend or acquaintance”), and employs a Likert scale with four points, from 1 (never) to 4 (frequently). Responses are summed, accounting for reverse-scored items, with higher scores reflecting higher loneliness. Although the SLS was originally developed as a general measure of loneliness, its items predominantly assess perceived deficiencies in social connections and interactions, which aligns with the concept of social loneliness operationalized in this study. In Chinese adolescent samples, the SLS has demonstrated strong validity and reliability (Zhou et al., 2026). Reliability analysis showed the scale to have acceptable internal consistency (Cronbach’s *α* = 0.816). The dataset was appropriate for factor analysis, with KMO = 0.859 and a significant Bartlett’s test (*p* < 0.001).

#### 3.2.2 Motives for online gaming questionnaire (MOGQ)

This research utilized the Escape Motivation subscale of the MOGQ developed by Demetrovics et al. (2011) to measure adolescents’ EM. The subscale includes four items rated on a 5-point Likert scale (1 = never, 5 = always), with higher scores reflecting stronger EM. Research has indicated that the subscale demonstrates satisfactory reliability and validity in Chinese populations (Gu and Mao, 2023). With a Cronbach’s *α* of 0.869, the subscale in this study demonstrated good internal reliability. KMO of 0.740 and a significant Bartlett’s test (*p* < 0.001) confirmed the data were appropriate for factor analysis.

#### 3.2.3 Self-control scale (SCS)

This research employed the SCS developed by Tangney et al. (2004) to assess adolescents’ levels of SC. The scale consists of 19 items covering five dimensions: impulse control, resistance to temptation, focus on study, healthy habits, and moderate entertainment. Fifteen reverse-coded items are scored on a 5-point Likert scale (1 = completely true, 5 = completely untrue), with higher scores representing higher SC. Among Chinese adolescents, the SCS shows good reliability and validity (Xie et al., 2026). Reliability analysis indicated the scale had excellent internal consistency (Cronbach’s α = 0.900). KMO (0.931) and Bartlett’s test (*p* < 0.001) confirmed suitability for factor analysis.

#### 3.2.4 Internet gaming addiction scale (IGAS)

This research used the IGAS developed by Charlton and Danforth (2007) to assess adolescents’ IGA. The 13-item scale covers four dimensions: personal conflict (4), withdrawal (3), relapse (3), and behavioral salience (3). Responses are rated on a 7-point Likert scale (1 = strongly disagree, 7 = strongly agree), with higher scores indicating greater IGA severity. Evidence from previous research indicates that the IGAS demonstrates satisfactory reliability and validity among Chinese adolescents (Wang and Zhang, 2026). The scale showed high reliability (Cronbach’s α = 0.946), with KMO = 0.947 and a significant Bartlett’s test (*p* < 0.001), confirming factor analysis suitability.

## 4 Statistical analysis

IBM SPSS version 27.0 was used for statistical analyses. Internal consistency and construct validity were assessed using Cronbach’s α, CR, and AVE, and descriptive statistics and normality tests were performed for all study variables. Potential common method bias (CMB) was first examined via Harman’s single-factor test and further evaluated using confirmatory factor analysis (CFA) and the unmeasured latent method construct (ULMC) approach in AMOS. AMOS was employed solely to assess the fit of the four-factor measurement model and compare it with a method factor model, and was not used to estimate the mediation model.

Pearson correlations were then calculated to examine associations among SL, EM, SC, and IGA. Parallel mediation was tested using the SPSS PROCESS macro (Model 4), with SL as the independent variable, EM and SC as parallel mediators, and IGA as the dependent variable, controlling for gender (dummy coded as 1 = male, 2 = female). Indirect effects were estimated via 5,000 bootstrap samples, with 95% bias-corrected CIs used to assess significance (Preacher and Hayes, 2008). The *R*^2^ values for SL → EM and SL → SC were 0.160 and 0.201, indicating moderate effect sizes. Although a formal power analysis was not conducted, these results suggest the indirect effects are meaningful. Because PROCESS estimates paths from observed scores, measurement error was not directly modeled; CFA results provide preliminary support for the reliability and validity of the measures.

## 5 Results

### 5.1 Descriptive statistics

Normality was assessed using skewness and kurtosis to ensure the data met multivariate analysis assumptions and to support the reliability of statistical tests. As noted by Kline (2015), Values of skewness below an absolute value of 2 and kurtosis under 7 can be considered indicative of normality. The skewness and kurtosis indices for the main variables in this research fell within acceptable ranges, indicating that the data generally adhered to the normal distribution pattern (see Table 3).

**Table 3:** Descriptive statistics.

Variables	*N*	M ± SD	SK	Kur
SL	731	16.343 ± 4.558	0.370	0.271
EM	731	8.287 ± 3.329	0.541	0.211
SC	731	64.118 ± 11.834	0.306	0.301
IGA	731	32.878 ± 15.369	0.427	−0.437

*N*, sample size; M, the means; SD, standard deviations; SK, skewness; Kur, kurtosis; SL, social loneliness; EM, escape motivation; SC, self-control; IGA, internet gaming addiction.

### 5.2 Composite reliability and validity

The outcomes of the reliability and validity assessments for all constructs are displayed in Table 4. According to Fornell and Larcker (1981), CR values greater than 0.70 indicate acceptable reliability, and Cronbach’s *α* values above 0.70 are considered satisfactory (Hayes, 2018). All constructs had CR and Cronbach’s α above the recommended thresholds (Table 4), indicating reliable internal consistency. Convergent validity was then assessed using the AVE, with values above 0.50 considered acceptable (Hayes, 2018). The results show that the AVE values for all constructs exceeded 0.50, suggesting good convergence among the measurement indicators.

**Table 4:** Reliability and convergent validity.

Constructs	CR	Cronbach’s α	AVE
SL	0.889	0.816	0.504
EM	0.865	0.869	0.622
SC	0.952	0.900	0.565
IGA	0.942	0.946	0.558

### 5.3 Common method bias and confirmatory factor analysis

Harman’s single-factor test was initially used to evaluate CMB. The first component explained 35.48% of the total variance, according to the data, which was less than the widely accepted cutoff of 40% (Podsakoff et al., 2003). However, because Harman’s single-factor test has been criticized as insufficient when used alone, a CFA-based ULMC approach was further conducted in AMOS.

Because the present study included four key latent variables, namely SL, EM, SC, and IGA, a four-factor CFA model was first constructed, with the items of each scale loading onto their corresponding latent factor. As shown in Table 5, the baseline four-factor model showed acceptable fit. This baseline model was then supplemented with a shared latent method factor, enabling all observed items to load on both the method factor and their theoretical constructs. The method factor model demonstrated satisfactory fit, with minimal differences in fit indices compared to the baseline model, all below recommended thresholds (Marsh and Hocevar, 1985). These data suggest that the results are unlikely to have been substantially impacted by common technique bias.

**Table 5:** Fit statistics for CFA and comparison of baseline and ULMC models.

Fit index	Reference value	Final model	Method factor model	Model fit difference	Evaluation criterion
CMIN/DF	<5	2.456	2.444		
RMSEA	<0.080	0.045	0.044	0.001	<0.05
GFI	>0.850	0.879	0.881	0.002	<0.01
AGFI	>0.850	0.854	0.855	0.001	<0.01
CFI	>0.900	0.941	0.942	0.001	<0.01
IFI	>0.900	0.942	0.942	0.000	<0.01
TLI	>0.900	0.932	0.933	0.001	<0.01

CMIN, chi-square value; DF, degree of freedom; RMSEA, root mean square error of approximation; GFI, goodness-of-fit index; AGFI, adjusted goodness-of-fit index; CFI, comparative fit index; IFI, incremental fit index; TLI, tucker-lewis index.

Furthermore, multi-group CFA was used to investigate if the measurement model was gender-neutral. Given the relatively large number of indicators, configural invariance was tested. The configural model showed acceptable fit (*χ*^2^ = 3251.038, df = 1,646, *χ*^2^/df = 1.975, CFI = 0.923, RMSEA = 0.037), suggesting that the overall factor structure was similar across male and female groups.

### 5.4 Collinearity diagnostics

To ensure the stability of the model and the validity of its results, multicollinearity was assessed. All variables had tolerance above 0.1 and VIF below 3.3, indicating no multicollinearity issues (Table 6) (Hair et al., 2019).

**Table 6:** Multicollinearity assessment for the structural model.

Variable	Tolerance	VIF
SL	0.773	1.293
EM	0.696	1.437
SC	0.649	1.542

SL, social loneliness; EM, escape motivation; SC, self-control.

### 5.5 Correlations among study variable

This study conducted correlation analyses to examine the relationships among SL, EM, SC, and IGA. As shown in Table 7, SL was positively correlated with EM (*r* = 0.376, *p* < 0.001) and IGA (*r* = 0.354, *p* < 0.001), and negatively correlated with SC (*r* = −0.447, *p* < 0.001). IGA was strongly positively associated with EM (*r* = 0.744, *p* < 0.001) and negatively with SC (*r* = −0.625, *p* < 0.001). Therefore, H1 is supported.

**Table 7:** Correlation analysis of variables.

Variable	1	2	3	4
SL	1			
EM	0.376^***^	1		
SC	−0.447^***^	−0.529^***^	1	
IGA	0.354^***^	0.744^***^	−0.625^***^	1

SL, social loneliness; EM, escape motivation; SC, self-control; IGA, internet gaming addiction; ^***^p < 0.001.

### 5.6 Mediation effect analysis

**Figure 2:**
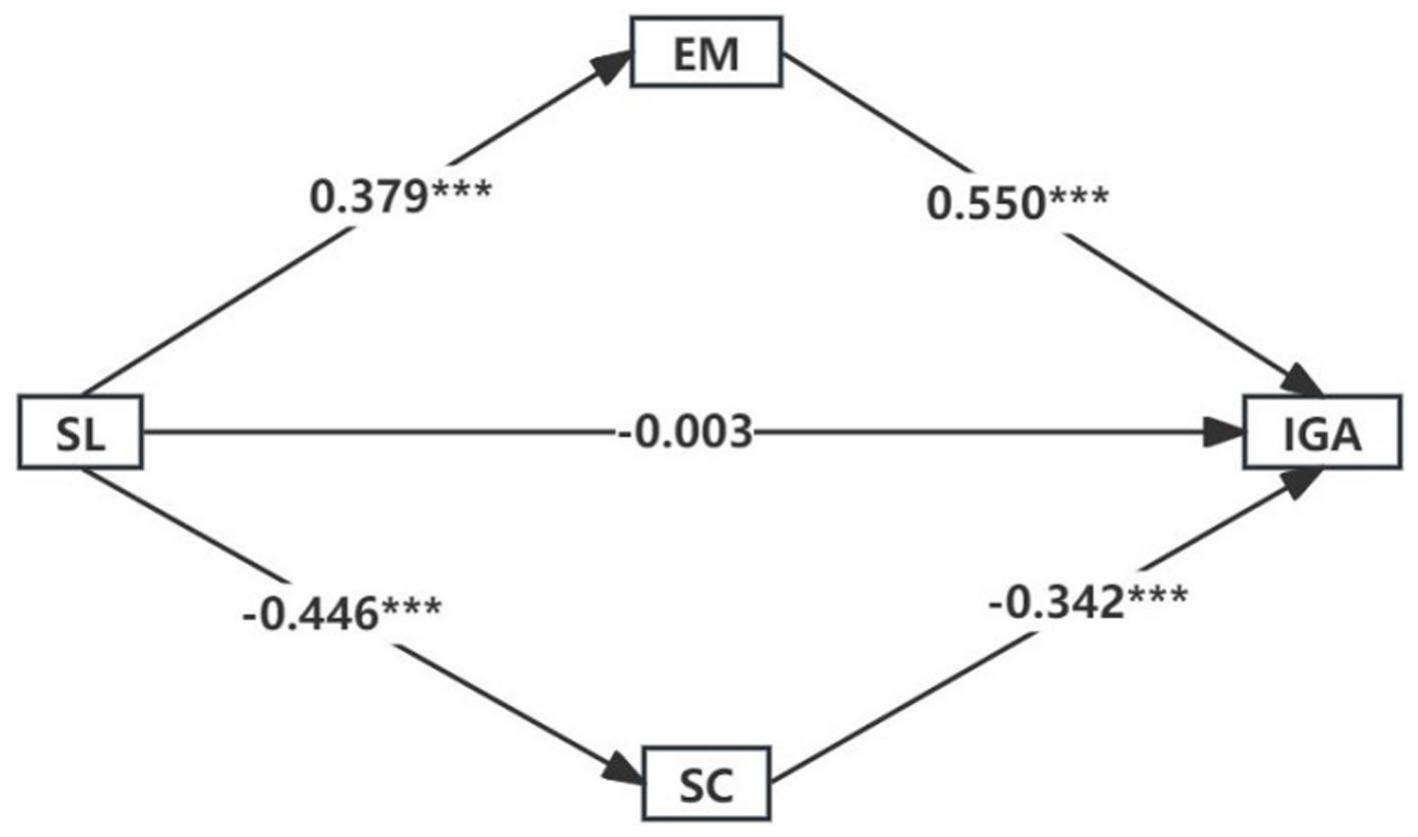
Mediation model diagram.

After controlling for gender, mediation analyses were performed using PROCESS Model 4. Gender was dummy coded (1 = male, 2 = female) and included as a covariate in the PROCESS mediation model. As shown in Table 8 and Figure 2, SL was positively associated with EM (*β* = 0.379, *p* < 0.001) and negatively associated with SC (*β* = −0.446, *p* < 0.001). EM was positively associated with IGA (*β* = 0.550, *p* < 0.001), while SC was negatively associated with IGA (*β* = −0.342, *p* < 0.001). The direct path from SL to IGA was not significant (*β* = −0.003, 95% CI [−0.053, 0.046]), whereas the indirect effects through EM (*β* = 0.208, 95% CI [0.161, 0.261]) and SC (*β* = 0.152, 95% CI [0.116, 0.191]) were significant, indicating that these mediators jointly accounted for the association between SL and IGA. The *R*^2^ values for SL → EM and SL → SC were 0.160 and 0.201, respectively, indicating moderate effect sizes. These results suggest that the identified mediating mechanisms have meaningful practical implications, as interventions targeting EM and SC could substantially reduce the risk of adolescent IGA.

**Table 8:** Mediation model regression results.

Predictor variables	Outcome variables	*β(std)*	*SE*	*t*	Bootstrap 95% CI		*R* ^2^	*F*
					LLCI	ULCI		
SL	EM	0.379^***^	0.034	11.144	0.312	0.445	0.160	69.275
Gender		−0.276^***^	0.069	−4.012	−0.412	−0.141		
SL	SC	−0.446^***^	0.033	−13.467	−0.511	−0.381	0.201	91.749
Gender		−0.080	0.067	−1.190	−0.212	0.052		
SL	IGA	−0.003	0.025	−0.127	−0.053	0.046	0.641	324.136
EM		0.550^***^	0.027	20.264	0.496	0.603		
SC		−0.342^***^	0.028	−12.282	−0.396	−0.287		
Gender		−0.235^***^	0.046	−5.116	−0.325	−0.145		

Gender was dummy coded as 1 = male and 2 = female and entered into the covariate field in the PROCESS macro. SL, social loneliness; EM, escape motivation; SC, self-control; IGA, internet gaming addiction; ^***^p < 0.001.

As shown in Table 9 and Figure 2, the total effect of SL on IGA was significant (*β* = 0.357, 95% CI = [0.291, 0.424]), indicating a positive association between SL and IGA. The direct effect of SL on IGA was not significant (*β* = −0.003, 95% CI = [−0.053, 0.047]), showing the relationship is fully mediated through EM and SC. Both parallel indirect paths were significant: the indirect effect through EM was 0.208 (95% CI = [0.161, 0.258]), accounting for 58.26% of the total effect, whereas the indirect effect through SC was 0.152 (95% CI = [0.117, 0.192]), accounting for 42.58% of the total effect. Moreover, the indirect effect through EM was larger than that through SC. Therefore, H2 and H3 were supported.

**Table 9:** Mediation effects of EM and SC in the association between SL and IGA.

Effect	Path	*β*	Bootstrap 95% CI		Percentage (%)
			LLCI	ULCI	
Total effect	SL → IGA	0.357	0.291	0.424	100.00
Direct effect	SL → IGA	−0.003	−0.053	0.047	−0.84
Indirect effect	SL → EM → IGA	0.208	0.161	0.258	58.26
	SL → SC → IGA	0.152	0.117	0.192	42.58

## 6 Discussion

As hypothesized, SL was indirectly related to IGA through EM and SC, while the direct association between SL and IGA was non-significant. Specifically, adolescents experiencing higher SL reported higher levels of EM and lower levels of SC, which were in turn associated with greater engagement in problematic gaming. These findings support the full mediation model and align with the CIUT, highlighting EM and SC as two distinct but complementary internal pathways linking SL to adolescent IGA.

### 6.1 Social loneliness and internet gaming addiction

The findings of this research indicated that SL strongly and positively forecasted IGA in adolescents, supporting H1. This outcome agrees with the research of Yu et al. (2022), indicating that individuals with higher SL tend to use online gaming to fulfill these needs, increasing their susceptibility to gaming addiction. As stated in the CIUT, Internet gaming provides individuals with an important means of coping with real-life stress, alleviating negative emotions, and compensating for unmet social needs (Melodia et al., 2022). Immersed in the virtual world of Internet games, players fulfill basic psychological needs—such as identity expression, social connection, and creativity—through teamwork, achievement, and other in-game activities (Giardina et al., 2021). However, this compensatory fulfillment carries potential negative consequences. Although adolescents may temporarily relieve feelings of loneliness through Internet gaming, the underlying causes of their SL remain unaddressed. In fact, Internet gaming may replace activities necessary for maintaining healthy relationships or forming new ones, thereby exacerbating their SL over time (Sporcic and Glavak-Tkalic, 2023). Therefore, the outcomes of this investigation highlight the critical role of SL in the development of IGA among adolescents and further validate the applicability of the CIUT in this context. These results suggest that school-based mental health programs should focus on adolescents’ social well-being, foster supportive social environments, and help meet adolescents’ psychological needs in real life, thereby addressing the root causes and ultimately reducing the incidence of IGA.

### 6.2 The mediating role of escape motivation

EM served as a mediator between SL and IGA among adolescents, supporting H2. The direct effect of SL on IGA was non-significant (*β* = −0.003, 95% CI [−0.053, 0.046]) when EM was included, suggesting full mediation. This finding suggests that the effect of SL on IGA operates entirely through EM, highlighting the central role of EM as the internal mechanism linking SL to addictive gaming behaviors. From the perspective of the CIUT framework, persistent SL as a negative emotional experience reinforces a tendency to avoid real-life stressors, prompting adolescents to use Internet gaming as a compensatory strategy to alleviate negative emotions (Kardefelt-Winther, 2014). SL drives adolescents to use the Internet for entertainment, seeking an escape from reality and a way to reduce their feelings of loneliness (Gabbiadini et al., 2021; Killgore et al., 2020). In this process, adolescents who frequently turn to gaming to avoid real-world challenges may develop cognitive distortions related to gaming, which further contributes to IGA (Xu et al., 2025). Moreover, heightened EM can lead to adolescents’ disengagement from real-world social activities, further increasing their risk of IGA (Zhen et al., 2019). The disappearance of the direct path indicates that the compensatory use of Internet gaming fully accounts for the effect of SL on IGA. This mechanism is consistent with prior research showing that EM is a key factor in problematic gaming behaviors (Xu et al., 2025), while extending previous work by demonstrating full mediation of SL on IGA through EM in a sample of adolescents. In summary, this research highlights an internal pathway linking SL and adolescent IGA through EM. The full mediation emphasizes that interventions aiming to reduce IGA in socially lonely adolescents should target EM directly, rather than attempting to reduce SL alone. Educational institutions and parents ought to provide support for adolescents in improving their social skills and expanding their social networks, thereby diminishing reliance on gaming as an escape or compensatory behavior.

### 6.3 The mediating role of self-control

The research indicated that SC acted as a mediator in the connection between SL and IGA in adolescents, supporting H3. Importantly, the direct path from SL to IGA became non-significant when SC was included in the model, indicating full mediation. This finding suggests that the effect of SL on IGA operates entirely through deficits in SC, highlighting self-regulatory processes as the key internal mechanism linking SL to addictive gaming behaviors. From the perspective of the CIUT framework, SL generates negative emotional experiences that deplete self-regulatory resources, making adolescents more susceptible to compensatory Internet gaming (Kardefelt-Winther, 2014). Adolescents with low SC find it difficult to regulate their Internet use rationally and are more likely to become excessively immersed in the pleasures associated with online activities (Quancai et al., 2023). The full mediation through SC indicates that deficits in self-regulatory capacity entirely explain the link between SL and IGA. This result aligns with prior research showing that lower SC is associated with maladaptive gaming behaviors in adolescent populations (Hu et al., 2025; Xiang et al., 2022), while extending previous work by providing direct evidence of full mediation in a younger sample. From a CIUT perspective, social loneliness-induced emotional depletion undermines SC, which fully accounts for the observed association with IGA. In summary, this study reveals the internal pathway through which SL increases adolescent IGA by weakening SC. The finding of full mediation underscores that interventions should prioritize strengthening adolescents’ SC abilities, enhance impulse inhibition, and build psychological resources to resist online temptations, rather than solely focusing on reducing SL.

## 7 Implications and limitations

### 7.1 Theoretical implications

Grounded in the CIUT, this study systematically constructed and validated a structural model linking SL, IGA, EM, and SC. By incorporating SL, a key psychological variable, into the framework of the CIUT to explain the mechanisms underlying adolescent IGA, this study broadens the theory’s scope beyond its traditional emphasis on social environmental factors like family and peers. The findings illuminate the internal connections through which psychological needs arising from social deprivation are compensated for via Internet gaming, thereby expanding the application of the CIUT in the context of Internet addiction research and enriching perspectives on the study of IGA.

In addition, this study confirmed the significant mediating roles of EM and SC in the association between SL and IGA, highlighting the roles of internal motivation and behavioral regulation in adolescent IGA. By validating these mediating pathways, the study provides a more refined model for understanding the psychological processes underlying addictive behaviors.

In sum, this research advances our theoretical comprehension of the underlying causes of IGA and stresses the necessity for future studies to examine how individuals’ internal psychological processes affect their behavior, thereby advancing interdisciplinary integration in the fields of Internet use behaviors and mental health.

### 7.2 Practical implications

The findings offer practical implications for addressing adolescent IGA by focusing on the mediating mechanisms of EM and SC, in light of the full mediation results.

First, at the school level, the findings indicate that interventions should focus on reducing escape-motivated gaming and enhancing SC rather than attempting to reduce gaming solely by addressing SL. Because SL influences IGA entirely through EM and SC, efforts that directly target these mediating mechanisms—such as structured peer interactions to reduce reliance on gaming as an escape, and digital literacy programs to develop healthier coping strategies—are expected to be most effective. Schools may provide more opportunities for peer interaction through sports, arts, science clubs, and other structured extracurricular activities, which can help adolescents reduce escape-motivated gaming. Teachers may also benefit from basic training to recognize signs of problematic gaming and loss of SC, so that timely support can be provided.

Second, at the family level, interventions should focus on enhancing adolescents’ SC and reducing escape-oriented gaming motives. Parents can support adolescents by helping them establish reasonable routines for screen use, study, rest, and recreation, and by reinforcing self-regulation skills in daily contexts. Efforts should target the mechanisms through which SL leads to IGA, rather than attempting to directly reduce loneliness itself.

Finally, at the individual level, adolescents may benefit from strategies that strengthen SC and reduce escape-oriented gaming motives. For example, they may be encouraged to set realistic limits on gaming time, use self-monitoring tools, participate in offline activities, and seek peer or adult support when feeling socially isolated. Since SL does not exert a direct effect on IGA, interventions aimed at reducing escapism-driven gaming and enhancing self-regulation are crucial, as these are the mechanisms through which SL increases gaming addiction risk. Prevention and intervention efforts should therefore place greater emphasis on targeting EM and SC, rather than focusing only on gaming frequency itself.

### 7.3 Limitations

This research made notable strides in examining the links between SL, EM, SC, and IGA. Nonetheless, certain limitations must be acknowledged, and additional studies are required to tackle these concerns.

First, this study utilized a cross-sectional research design. Although the CIUT provided a theoretical basis for the directional assumptions of the variable relationships, and the study tested the mediating effects, causal relationships between variables could not be established. Long-term research designs are needed in future studies to monitor how these variables change over time and uncover the cause-and-effect relationships between them.

Second, self-reported data may be affected by social desirability and recall biases. While CFA supported the measurement model, PROCESS estimates paths using observed composite scores and does not account for measurement error. Future research could use full SEM to simultaneously estimate measurement and structural models, and incorporate multi-source data to improve reliability. In addition, although a formal power analysis for the mediation effects was not conducted, the moderate *R*^2^ values for SL → EM (0.160) and SL → SC (0.201) and significant bootstrap CIs suggest the observed indirect effects are meaningful. Simulation-based power analyses could further complement these results in future studies.

Third, this study focused on adolescents in China. While most scales (SLS, SCS, and IGAS) have been validated in Chinese adolescent populations, the EM subscale of the MOGQ has only been validated in Chinese university students, which may limit cross-cultural measurement equivalence. Additionally, given that the SLS was originally developed as a general loneliness measure, it may not fully capture the conceptual distinction between social and emotional loneliness, which could affect the specificity of the observed relationships. Future research could employ instruments that explicitly distinguish social from emotional loneliness, and validate the EM subscale among Chinese adolescents to enhance measurement precision and cross-cultural generalizability. Moreover, to further improve the generalizability and cultural relevance of the findings, future studies should incorporate adolescents from diverse cultural contexts. Although configural invariance across gender was supported, stricter forms of measurement invariance were not fully examined. Future studies could further test metric and scalar invariance across gender to strengthen comparability between male and female adolescents.

## 8 Conclusion

Grounded in the CIUT, this research identified systematic pathways linking SL and IGA among adolescents. The findings indicated that EM and SC fully mediated SL’s association with IGA. This research advances the theoretical understanding of adolescent IGA by exploring the factors at play and providing deeper insights into the internal processes related to EM and SC, offering new directions for future research. In sum, this study offers theoretical support for the link between SL and IGA and provides insights for preventing and addressing adolescent IG, while highlighting potential avenues for future investigation.

## Data Availability

The datasets presented in this study can be found in online repositories. The names of the repository/repositories and accession number(s) can be found in the article/supplementary material.

## Footnotes

1
https://www.wjx.cn/
